# Comparison of the lower genital tract microbiome composition in patients with benign gynecological disease

**DOI:** 10.3389/fgwh.2025.1507907

**Published:** 2025-01-21

**Authors:** Yonghui Shi, Jun Li, Jinjing Xie, Tianye Yang, Qiongyan Ma, Hua Chen, Wenwei Guo

**Affiliations:** ^1^ Department of Obstetrics and Gynecology, Gongli Hospital, The Second Military Medical University, Shanghai, China; ^2^ Department of Obstetrics and Gynecology, Taiping Street Community Health Service Center, Suzhou, China

**Keywords:** endometrial polyp, uterine myoma, ovarian cyst, microbiota dysbiosis, 16S rRNA sequencing

## Abstract

**Objective:**

Lower genital tract microbiome dysbiosis has been associated with several gynecological diseases. However, the differences in microbiome composition among patients with several gynecological diseases, such as endometrial polyps and uterine myoma, are poorly understood. Studying the lower genital tract microbiome composition in patients with benign gynecological diseases could provide new insights for interpreting the complex interplay between the microbiome and pathogenesis and finding new targets for preventive measures.

**Methods:**

A total of 16 patients with endometrial polyps (EPs), 11 patients with uterine myoma (UM), 6 patients with ovarian cysts (OC) and 36 healthy women (HWs) were recruited for this study. Samples were obtained from vaginal secretions. The DNA was isolated from the samples, and the V3-V4 regions were amplified. The sequencing libraries were generated and sequenced on an Illumina NovaSeq 6000 platform.

**Results:**

Firmicutes, Actinobacteria and Bacteroidota were the most common phyla in all four groups, whereas OC presented the highest abundance of Firmicutes and the lowest abundance of Bacteroidota. At the genus level, *Lactobacillus* in the OC group was significantly greater than that in the HW group, and *Atopobium* in the UM group was significantly lower than that in the HW group. The abundance of *Gardnerella* was greater in the UM group than in the EP group, and the abundance of *Streptococcus* was greater in the EP group. The richness and evenness of the microbiome were generally consistent among the HW, EP, UM, and OC groups. Principal component analysis (PCA), principal coordinate analysis (PCoA) and nonmetric multidimensional scaling (NMDS) revealed no distinct separation trends among the four groups. According to ANOSIM, there was no significant difference in community structure among the four groups.

**Conclusions:**

A nonsignificant result was obtained from the microbiome diversity comparison among the different groups. However, we demonstrated that the OC group had a greater abundance of *Lactobacillus* and that the UM group had a lower abundance of *Atopobium,* which might contribute to the occurrence of diseases, providing new clues for preventive measures.

## Introduction

The vaginal microbiome is an important part of the female reproductive system and is composed of a complex microbial community dominated by *Lactobacillus*. The health status of the vaginal microecology has an important impact on women's overall health and reproductive function (1, 2). Vaginal microecological imbalance can lead to a series of reproductive system diseases, such as bacterial vaginosis and vaginal candidiasis (3).

The vaginal microbiome is dominated by lactobacilli (such as *Lactobacillus crispatus* and *Lactobacillus iners*), which produce lactic acid, hydrogen peroxide, and other substances to maintain the acidic environment of the vagina and inhibit the growth of pathogenic microorganisms (1, 4). Other commensal bacteria, such as *Bifidobacterium* and *Staphylococcus epidermidis*, also play a supporting role in maintaining the vaginal microbial balance (5).

The vaginal microbiome can inhibit pathogenic microorganisms through competitive exclusion and the production of antimicrobial substances. It can also regulate local and systemic immune responses through interactions with the host immune system (6, 7). In addition, the vaginal microbiome can maintain the pH value of the vagina, promote a healthy reproductive environment, help prevent infections and promote fertility (8, 9).

In recent years, with the development of high-throughput sequencing technology, the study of the vaginal microbiome has made important progress, and its diversity is closely related to women's health status (3, 10). In addition to clearly causing female vaginal inflammation, this disorder is closely related to a variety of reproductive system diseases, such as cervical cancer and premature birth (11, 12). In addition to malignant tumors, many gynecological benign diseases, such as endometrial polyps, uterine fibroids, and ovarian cysts, can also affect women (12–15).

Endometrial polyp is a common gynecological disease, and its etiology is not completely clear. Endometrial polyps are focal intrauterine endometrial tumors that can be single or multiple. They range in size from a few millimeters to a few centimeters and may be sessile in form, with large or small implantation bases or pedicles (16). Endometrial polyps may be asymptomatic (17), and when they cause symptoms, the most common clinical manifestations include abnormal (including postmenopausal) uterine bleeding and infertility (18, 19). Malignant transformation occurs in 0–12.9% of cases (20) and severely affects female reproductive health.

Uterine fibroids are the most common benign tumors in the female reproductive system, the exact cause of which is not clear, and their incidence is high in the female population (21). The symptoms of uterine fibroids depend on the location of the fibroids. While not all fibroids have symptoms, typical symptoms include abnormal uterine bleeding/excessive menstrual volume (AUB/HMB), pelvic volume symptoms (abdominal protrusion, bladder and bowel pressure), pain, and reproductive complications (i.e., infertility) (22).

Ovarian cysts are also common benign lesions in the female reproductive system, and their etiology and pathogenesis are complex and diverse (23). Small ovarian cysts are usually asymptomatic, and larger cysts are often found in the acute abdomen due to rupture and torsion. These benign diseases are usually treated with surgery, but they are relatively prone to relapse (24). Exploring the possible pathogenesis of these diseases will help develop new prevention and treatment strategies to improve female reproductive health.

The purpose of this study was to analyze the composition and changes in the vaginal microbiota of patients with endometrial polyps, uterine fibroids and ovarian cysts via high-throughput 16S rRNA sequencing technology and to explore the relationships between vaginal microecology and these gynecological diseases. By comparing the diversity and composition of the microbiota between different groups, the potential mechanism of vaginal microbiota imbalance in gynecological diseases can be revealed, and new perspectives and methods can be provided for the prevention and treatment of these diseases.

## Methods

### Patients and specimen collection

In this study, 36 healthy women and 39 patients were recruited at the Gongli Hospital of the Second Military Medical University between January 2024 and March 2024. The selection criteria were as follows: (I) B-ultrasound did not suggest other gynecological diseases in healthy women, and vaginal secretions were not examined for inflammation; (II) patients were diagnosed with endometrial polyps, uterine fibroids, and ovarian cysts by B-ultrasonography without vaginitis and who were ready for surgical treatment; (III) all the subjects had not taken antibiotics for three months before surgery; and (IV) all the subjects had nonmenstrual periods and no abnormal uterine bleeding. The specimens were obtained from vaginal secretions, one-third of the vaginal wall was removed with a sterile swab brush, and the sterile swab was inserted into a sterile frozen tube containing 0.9% normal saline, immediately placed in liquid nitrogen for quick freezing and stored at −80°C. The clinical data of all participants, including age, body mass index (BMI), and pathology type, were collated.

The study was conducted in accordance with the Declaration of Helsinki. The study was approved by the ethics committee of the Gongli Hospital of the Second Military Medical University, Shanghai, China. All the subjects provided consent before participation.

### Genome DNA extraction and 16s rRNA gene amplicon sequencing

Total genomic DNA from the samples was extracted according to the manufacturer's protocols. The DNA concentration was monitored with a Qubit® dsDNA HS Assay Kit. The preparation of next-generation sequencing libraries and Illumina sequencing was conducted by the same company. The sequencing library was constructed via a MetaVX Library Preparation Kit. Briefly, 20–50 ng of DNA was used to generate amplicons that cover the V3 and V4 hypervariable regions of the 16S rRNA gene of bacteria. The forward primer sequence was “CCTACGGRRBGCASCAGKVRVGAAT”, and the reverse primer sequence was “GGACTACNVGGGTWTCTAATCC”. The concentration was detected via a microplate reader (Tecan, Infinite 200 Pro), and the fragment size, which is expected at ∼600 bp, was detected via 1.5% agarose gel electrophoresis. Next-generation sequencing was conducted on an Illumina MiSeq/Novaseq platform (Illumina, San Diego, USA) at the company. Automated cluster generation and 250/300 paired-end sequencing with dual reads were performed according to the manufacturer's instructions.

### Data and statistical analysis

Double-end sequencing of positive and negative reads with the first of the two joining together to filter together the results contained in the N sequence revealed that the sequence length was greater than 200 bp. After the quality filter, chimeric sequences were purified, and the resulting sequence for OTU clustering was subjected to VSEARCH clustering (1.9.6) (the sequence similarity was set to 97%). The 16S rRNA reference database used was Silva, 138. The RDP classifier (Ribosomal Database Program) Bayesian algorithm of OTU species taxonomy analysis was subsequently used to analyze representative sequences, and under different species classification levels, the community composition of each sample was statistically analyzed.

On the basis of the results of the OTU analysis, when the random sampling sample sequences were flat, the Shannon and Chao1 alpha diversity indices, community species abundance and diversity of rarefaction curves and rank–abundance graphs reflected the species richness and evenness.

UN-weighted UniFrac analysis was used to compare samples to determine whether there were significant differences in the microbial community. PCA, PCoA, and NMDS display beta diversity visualization, and PCA (principal component analysis) is based on the sample OTU abundance table. PCoA (principal coordinate analysis) and NMDS (nonmetric multidimensional scaling) are based on the distance between the Brary and Curtis matrices.

Anosim by comparing the differences between the analysis group rank values and rank within the group differences according to whether the group has significance. Metastats gap analysis involves the use of rigorous statistical methods to assess the differences in the abundances of the two groups of microbial community species.

### Predictive functional analyses

Microbial function prediction analysis was performed with PICRUSt software (v2.0.0). PICRUSt2 first compares the OTU sequence with its internal reference sequence, puts the OTU into the corresponding reference tree, inferences the copy number of each Gene family, predicts the gene content of each gene family, and determines the abundance of each sample gene family. Gene families will compare the information and the function of the corresponding database, including KEGG (https://www.kegg.jp/) and COG (https://www.ncbi.nlm.nih.gov/COG/) and so on, to get each sample of the function of the corresponding information belong degrees. KEGG database is a systematic analysis of gene function, contact genome information and function of large knowledge base. The KEGG GENES database provides sequence information on genes and proteins found in the genome project; The KEGG PATHWAY database includes various metabolic pathways, synthetic pathways, membrane transport, signaling, cell cycle, and disease-related pathways. In addition, information about various chemical molecules, enzymes and enzymatic reactions was collected.

## Results

### Patient characteristics at enrollment

A total of 36 healthy women and 39 patients were enrolled in this study from Gongli Hospital, and the final expanded sample included 36 healthy women and 33 patients. Table 1 describes the characteristics of the study population. The mean age was 44.5 years, and the mean BMI was 23.7. There were no statistically significant differences among the four groups.

**Table 1:** Characteristics of the study population.

Characteristics	Total (*n* = 69)	Healthy women (*n* = 36)	Endometrial polyp (*n* = 16)	Uterine myoma (*n* = 11)	Ovarian cyst (*n* = 6)	*P*
Age, years, mean (SD)	44.5 (7.8)	44.2 (7.7)	43.9 (8.2)	47.5 (4.4)	42.3 (12.3)	0.524
BMI, kg/m^2^, mean (SD)	23.7 (3.7)	23.3 (3.9)	23.6 (3.9)	25.6 (3.1)	22.3 (2.9)	0.253

### OTU analysis and species notes

**Figure 1:**
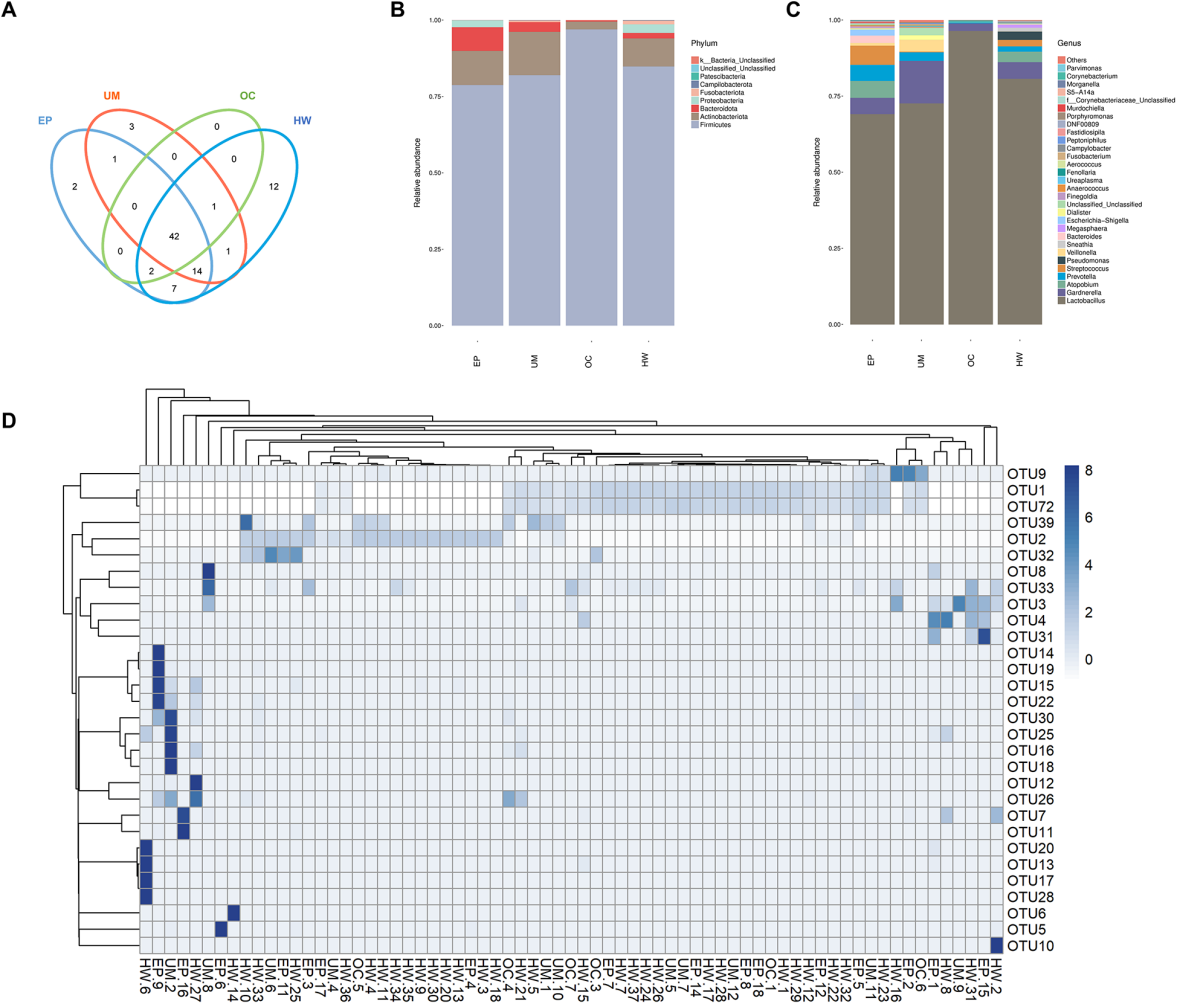
Operational taxonomic unit (oUT) analysis and species annotation in four groups (HW, EP, UM and OC). **(A)** Venn diagram: OTUs shared and unique to the four groups. **(B)** The relative frequency of the microbiome in each group at the phylum level. **(C)** The relative frequency of the microbiome in each group at the genus level. **(D)** Heatmap of the expression abundance of the top 30 OTUs of the microbiome in each sample. HW, healthy woman; EP, endometrial polyp; UM, uterine myoma; OC, ovarian cyst.

All the sequences obtained from the sequencing of all the samples were classified by OTUs at the 97% similarity level, and statistical analysis of the biological information was carried out. A total of 85 OTUs were obtained. Figure 1D shows the 30 OTU heatmaps with the highest abundance. The row name is the OTU ID, the column name is sample information, the OTU cluster tree is on the left of the figure, and the sample cluster tree is on the top. The value corresponding to the color of each square in the heatmap is the relative abundance of OTUs in each row after normalization. The figure shows that most samples are clustered in OTU1, OTU2 and OTU72. There was no significant difference.

In accordance with the results of the OTU clustering, the common and unique OTUs of the different groups were analyzed to obtain a Venn diagram (Figure 1A). The different colored circles in the Venn diagram represent different groups, and the numbers in the diagram represent the number of OTUs unique to each group or shared by each group. The results revealed 12, 2, 3 and 0 OTUs in the healthy female group, endometrial polyp group, uterine fibroid group and ovarian cyst group, respectively. The four groups had a total of 42 OTUs.

Figures 1B, C show the distributions of the top 30 species in each group at the phylum and genus levels, respectively. The horizontal coordinate represents the group name, and the vertical coordinate represents the relative abundance of different species. *Lactobacillus* was the main strain in all groups, and its expression abundance was the highest in the ovarian cyst group, while the expression abundance in the uterine myoma and endometrial polyp groups was lower than that in healthy women. The abundance of *Streptococcus* in patients with endometrial polyps was greater than that in the other three groups, and the abundance of *Gardnerella* in patients with uterine fibroids was greatest among the four groups.

### Bacterial alpha diversity among different groups

**Figure 2:**
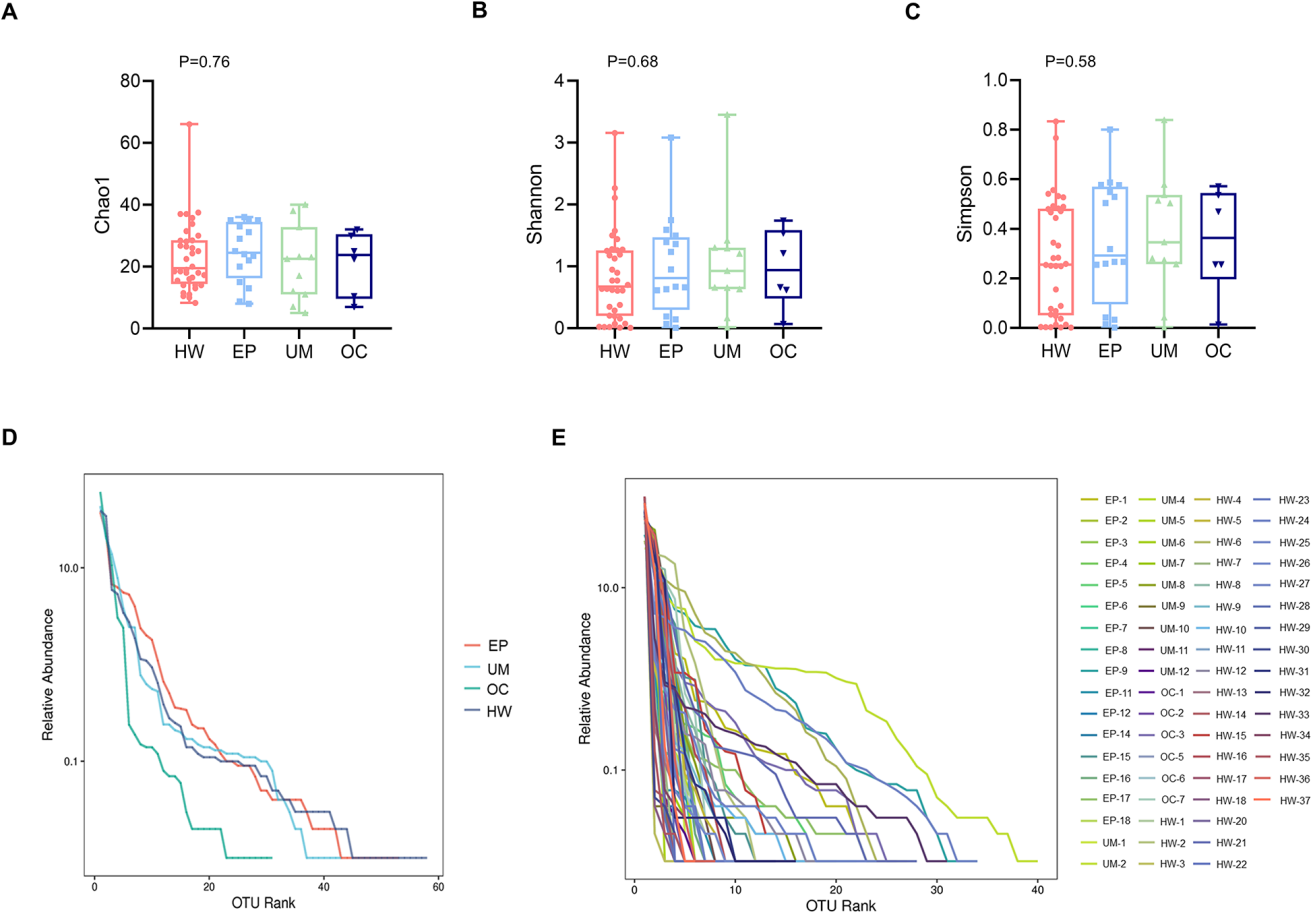
Comparison of the alpha diversity of the four groups. Alpha diversity, as indicated by the Chao1 **(A)**, Shannon **(B)** and Simpson **(C)** indices, was compared among the four groups. The *P* values were derived from the Wilcoxon rank sum test. **(D)** Rank abundance curve at the average level in each group. **(E)** Rank abundance curve at the individual level. HW, healthy woman; EP, endometrial polyp; UM, uterine myoma; OC, ovarian cyst.

The richness and evenness of the microbiome were generally consistent among the HW, EP, UM and OC groups, as estimated by the Chao 1 estimator (K-W, *P* = 0.76), Shannon index (K-W, *P* = 0.68), and Simpson index (K-W, *P* = 0.58) (Figures 2A–C). No significant differences were detected in any of the three indices among the four groups.

Figures 2D,E shows the rank‒abundance curve, which reflects species abundance and species evenness. Species abundance is reflected by the length of the curve on the horizontal axis. The larger the range of the curve on the horizontal axis is, the greater the species abundance. The flatter the curve is, the more homogeneous the species. Figure 2D shows that the rank abundance curves of the EP, UM, and HW groups appeared to be wider, suggesting greater bacterial richness in those groups. In addition, these groups presented a narrower vertical span in the rank abundance curve than the OC group did, indicating a more even distribution of bacterial composition, although the difference among the four groups was not significant.

### Beta diversity among different groups

**Figure 3:**
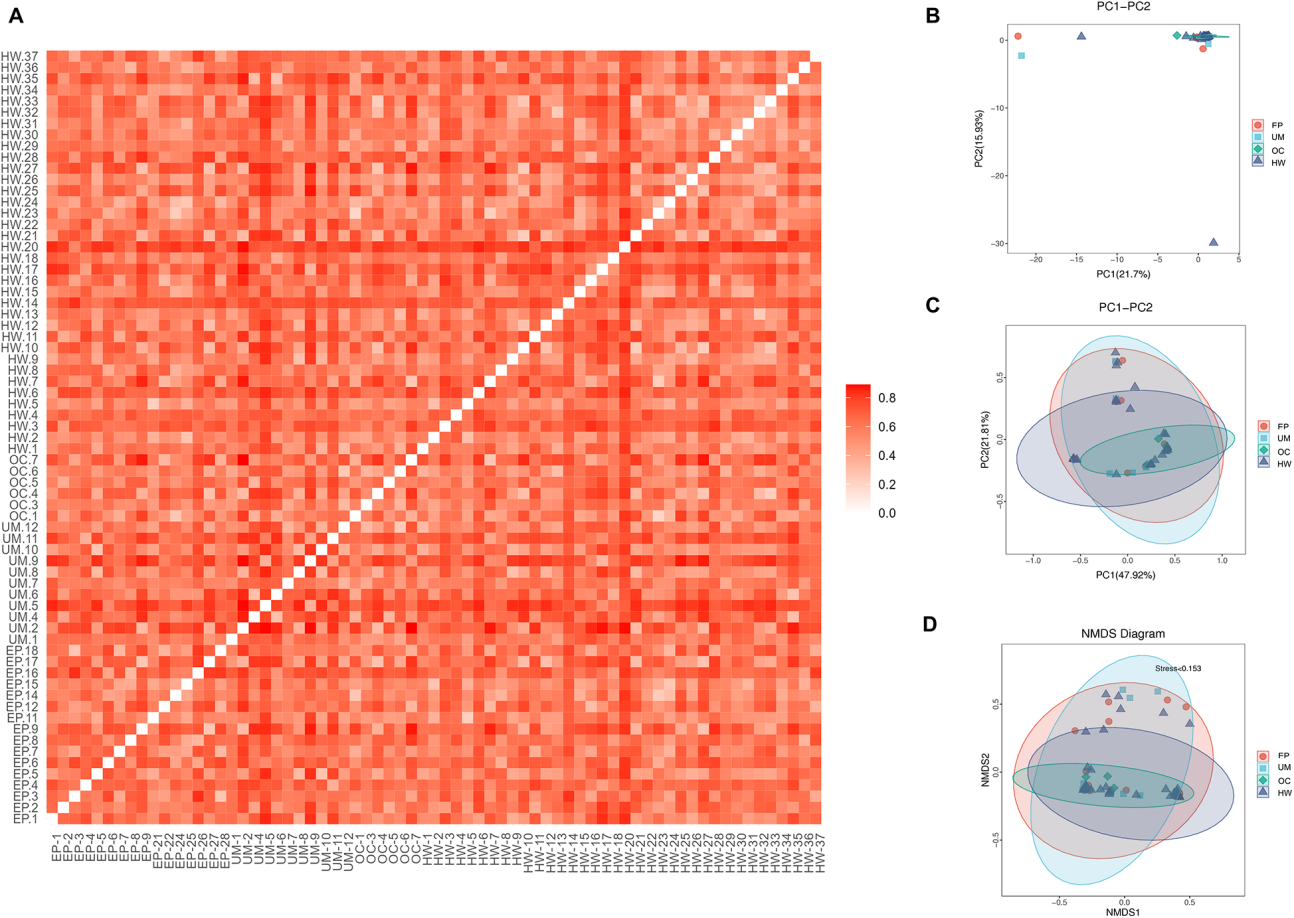
Comparison of beta diversity among the four groups. **(A)** Heatmap of evolution and abundance information among sample sequences. **(B)** Principal component analysis (PCA). **(C)** Principal coordinate analysis (PCoA). **(D)** Nonmetric multidimensional scaling analysis based on the beta diversity distance matrix. HW, healthy woman; EP, endometrial polyp; UM, uterine myoma; OC, ovarian cyst.

Figure 3 shows the beta diversity of the microbiome in the four sample groups. Figure 3A shows an unweighted UniFrac heatmap, in which the color shading represents the degree of difference between two samples. The lighter the color is, the smaller the coefficient of difference between the two samples, and the smaller the difference in species diversity. PCA, PCoA and NMDS analysis revealed no distinct separation trend among the four groups (Figures 2B–D). The distance between sample points in the figure represents the similarity of microbial communities in the samples. The closer the distance is, the greater the similarity, and the samples that are clustered together are composed of similar microbial communities.

### Analysis of significant differences in community structure between groups

A meta-analysis of the species composition at the genus level revealed that the abundance of *Lactobacillus* in the OC group was significantly greater than that in the healthy female group (Figure 3A, *P* < 0.05). The abundance of *Atopobium* in the UM group was significantly lower than that in the HW group (Figure 3B, *P* < 0.05). The abundance of *Streptococcus* in the EP group was greater than that in the HW group, but the difference was not statistically significant (Figure 3C, *P* > 0.05).

**Figure 4:**
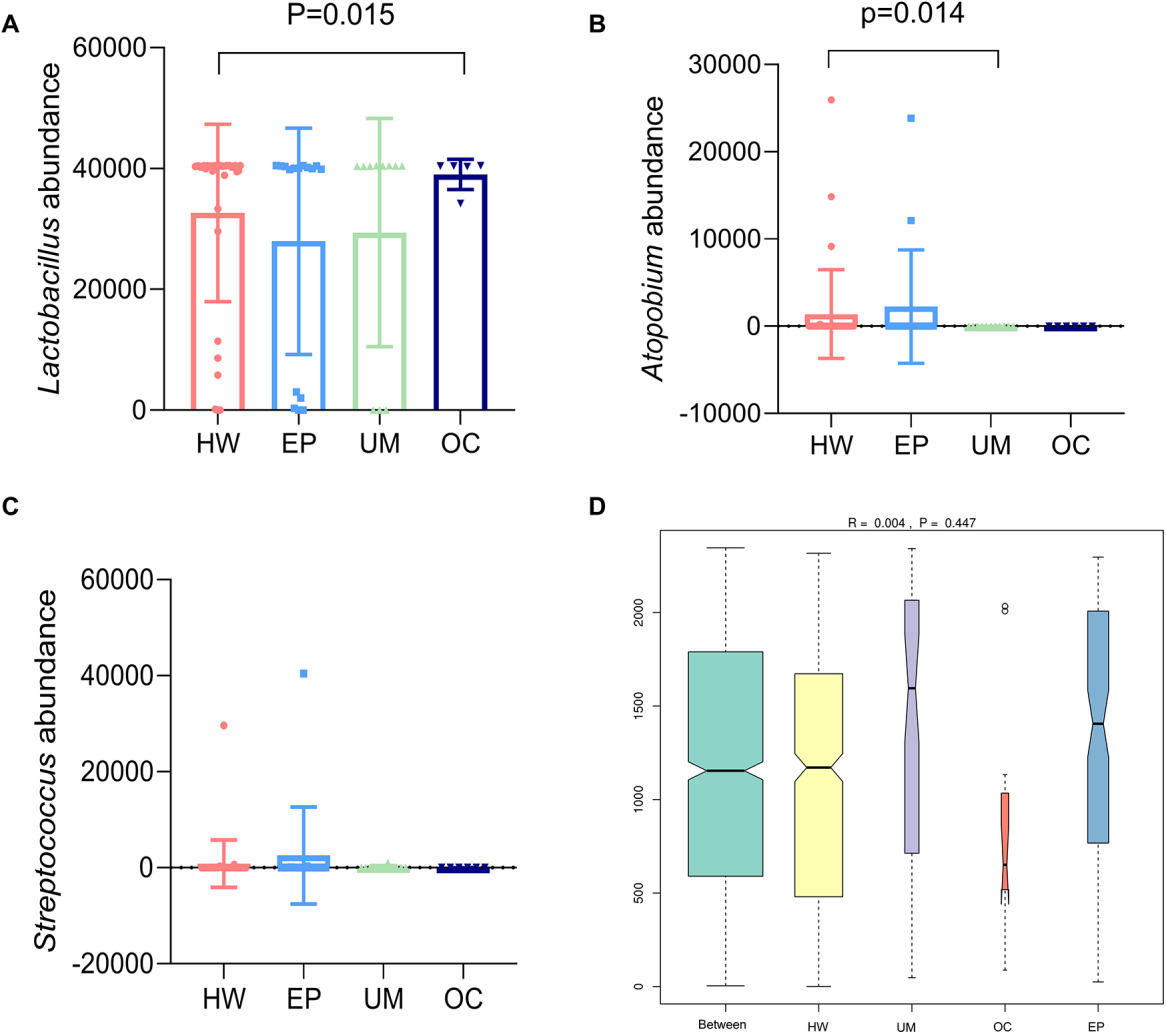
Analysis of significant differences in community structure between groups. The three species with large differences in community structure were abundant: **(A)** Lactobacillus, **(B)** Atopobium, and **(C)** Streptococcus. **(D)** ANOSIM among the four groups. HW, healthy woman; EP, endometrial polyp; UM, uterine myoma; OC, ovarian cyst.

ANOSIM is used to test whether the differences between groups are greater than the differences within groups. An R value close to 0 indicates that there are no significant differences between groups or within groups, and an R value close to 1 indicates that the differences between groups are greater than the differences within groups. Figure 4D shows that there were no significant differences between or within the four groups (*R* = 0.004, *P* = 0.447).

### Predictive functional analyses

**Figure 5:**
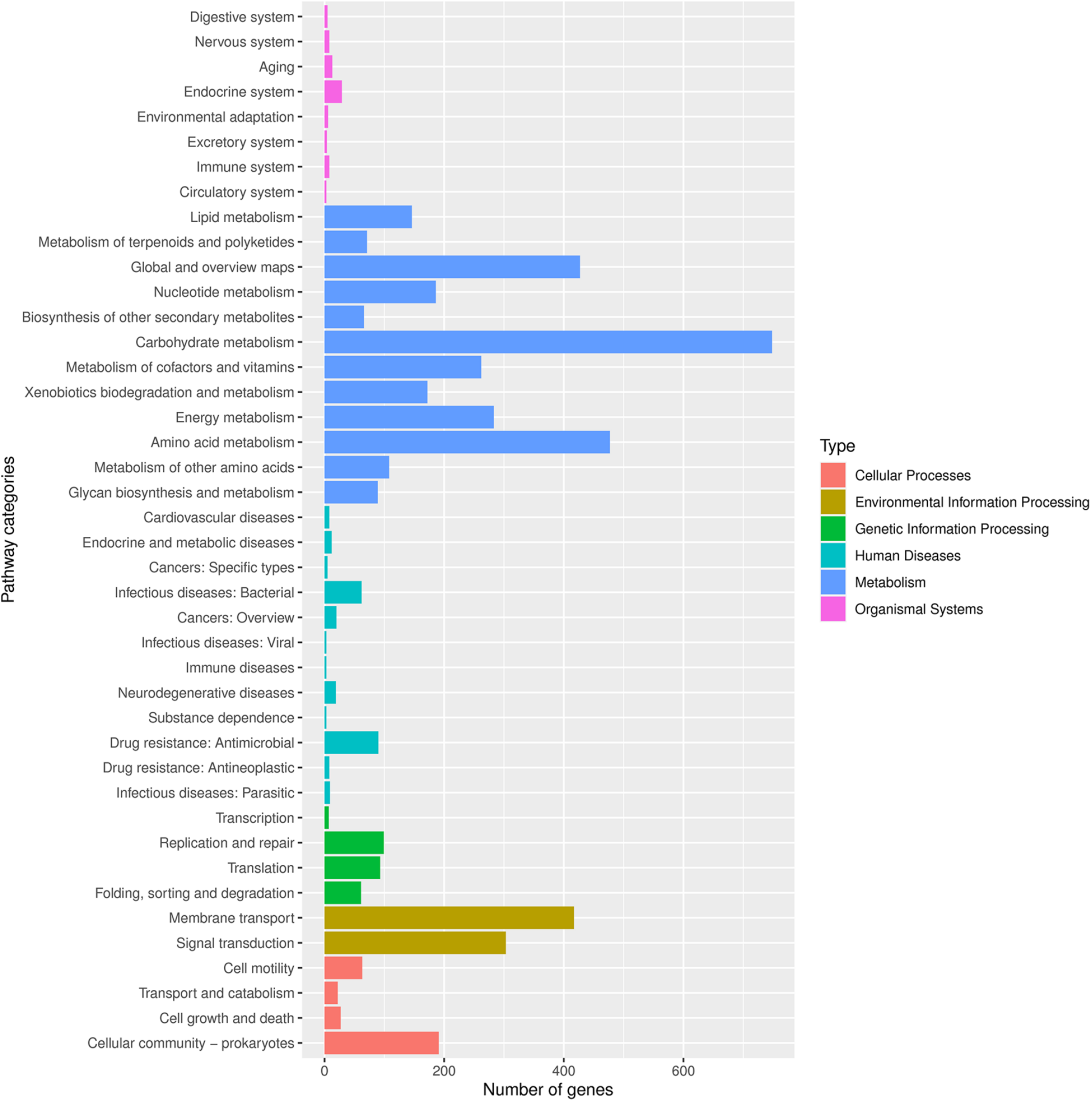
Number of genes associated with functional pathways. Different colors represent different types, as shown in the notes on the right side of the figure.

**Figure 6:**
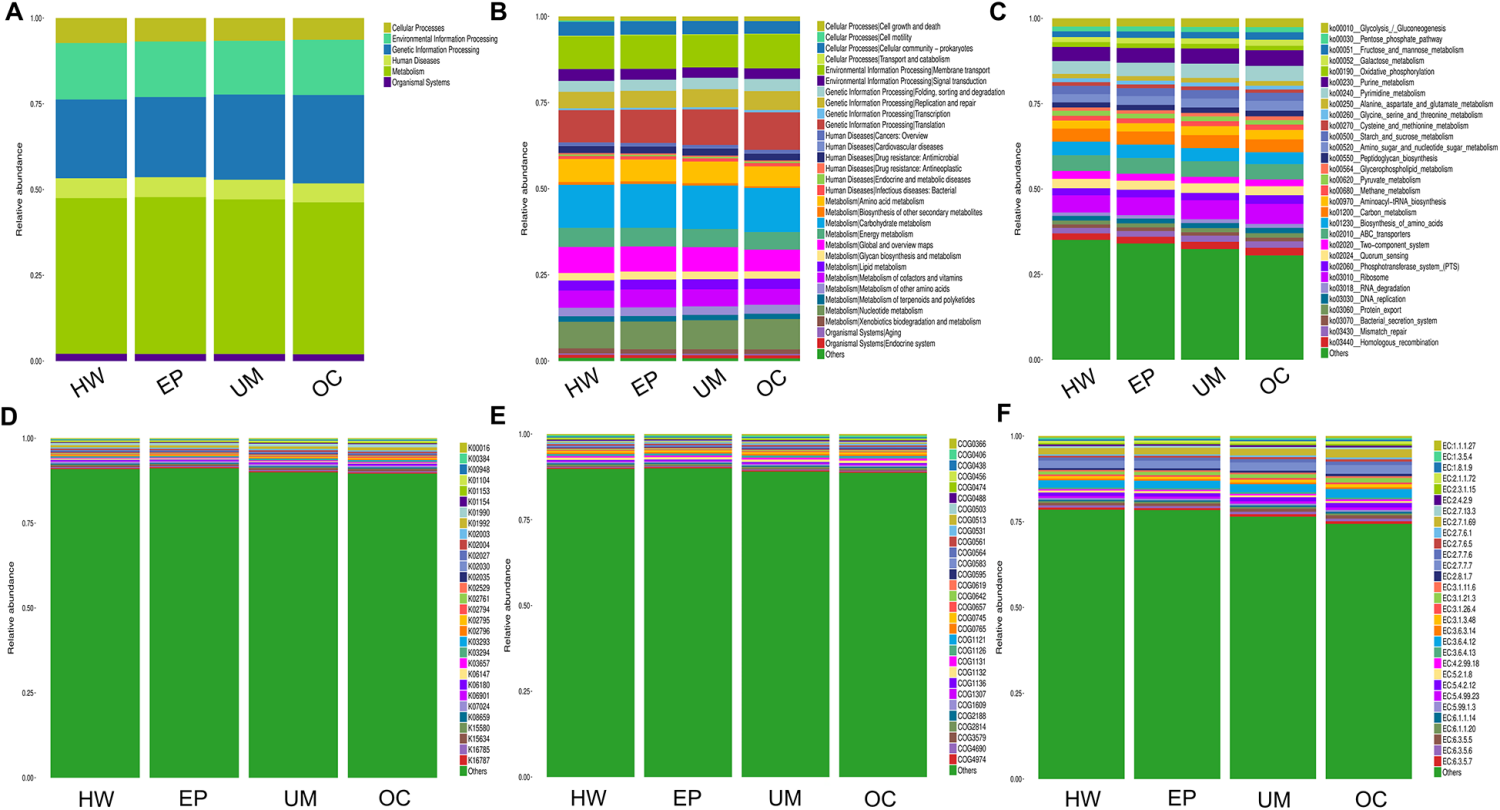
PICRUSt2 function prediction analysis. The relative abundance of each group in 6 major metabolic pathways **(A)**, 45 metabolic pathway subfunctions **(B)**, corresponding metabolic pathway map **(C)**, corresponding to each KEGG orthologous gene cluster in the metabolic pathway **(D)**, different COG categories **(E)** and enzyme function classes **(F).**

Finally, we conducted Phylogenetic Investigation of Communities by Reconstruction of Unobserved States (PICRUSt). The COG (Cluster of Orthologous Groups) analysis showed that the relative abundance distribution of different categories in each group was basically consistent, indicating that the COG composition patterns of different groups were very similar (Figure 6E). The KEGG (Kyoto Encyclopedia of Genes and Genomes) functional prediction indicated that the number of genes related to metabolic pathways was the largest (Figure 5), including glycolysis, amino acid metabolism, and carbon metabolism, showed certain differences between the groups, but the differences were not significant. In addition, the abundance of functions related to signal transduction, membrane transport, and other environmental information processing was also found to vary, especially in the group of ovarian cysts. This result suggests that the vaginal microbiome may affect the pathological process of different gynecological diseases by regulating metabolic functions and environmental information transmission (Figures 6A–F).

## Discussion

The vaginal microbiome is a complex ecosystem composed of a variety of microbes, of which the dominant microbiome in the vagina of healthy women is *Lactobacillus spp*. These *Lactobacilli* include *Lactobacillus crispatus*, *Lactobacillus jensenii*, *Lactobacillus gasseri* and *Lactobacillus iners,* which protect the vaginal environment through multiple mechanisms and play important roles in female reproductive health. They maintain the acidic environment of the vagina (pH approximately 4.5) by producing lactic acid, which inhibits the growth of pathogens. *Lactobacillus* can also produce hydrogen peroxide and bacteriocins, which have antibacterial activity and further enhance the defense against pathogenic microorganisms (25, 26).

Some benign gynecological diseases, such as endometrial polyps, uterine fibroids, and ovarian cysts, are treated mainly by surgery, but there is a possibility of recurrence after surgery. Because the pathogenesis of these diseases is not clear, there is no clear prevention method. In this study, healthy women and patients with endometrial polyps, uterine fibroids and ovarian cysts were recruited, and their vaginal secretions were subjected to 16S rRNA sequencing to analyze the vaginal microbiome. The results of the alpha and beta diversity analyses of the four groups revealed no significant differences in the diversity of the vaginal microbiome.

Our study revealed that the vaginal microbiota of all groups was dominated by *Lactobacillus*, but compared with that in the healthy female group, the abundance of *Lactobacillus* in the patients with ovarian cysts was significantly greater, and the difference was statistically significant (*P* < 0.05). The *Lactobacillus* abundance in the other three groups was lower than that in the healthy group, but the difference was not significant. This finding is consistent with reports in the literature suggesting a potential link between a decrease in vaginal *Lactobacillus* and the development of uterine fibroids and that low *Lactobacillus* levels in the vaginal microbiome increase inflammation and promote smooth muscle cell proliferation and extracellular matrix production, which may increase the risk of uterine fibroids (27).

In the context of vaginal microbiome disorders, the numbers of *Gardnerella* and other anaerobic bacteria (such as *Clostridium perfringens*) increase, and these bacteria can disrupt the normal ecological balance of the vagina, leading to infection and inflammation (25, 28). For example, studies have shown that *Gardnerella vaginalis* is strongly associated with the development of bacterial vaginosis, which in turn is associated with multiple reproductive health problems, including an increased risk of sexually transmitted diseases and preterm birth (28).

*Candida albicans* is a common yeast species in the vagina, and when it is overgrown, it can cause fungal vaginitis. Studies have shown that a healthy population of *Lactobacillus* can inhibit the growth of *Candida* and prevent its excessive proliferation (29). Inflammation plays a critical role in the development of uterine fibroids, characterized by elevated expression levels of pro-inflammatory and inflammatory cytokines such as interleukin-1, interleukin-6, interleukin-10, TNF-α, and TGF-*β*. These cytokines are essential mediators in the interaction between growth factors and the extracellular matrix. In women with uterine fibroids, the levels of pro-inflammatory mediators are notably elevated, with higher expression observed in the endometrium of those with submucosal and intermuscular fibroids compared to those without fibroids. This suggests a potential link between endometritis and uterine fibroids (30). Research has demonstrated significant differences in the uterine and vaginal microbiota between women with chronic endometritis and healthy controls. Women with the disease exhibit higher vaginal microbiota diversity, with notable changes including a reduced proportion of *Lactobacillus* and an increased prevalence of *Prevotella* and *Gardnerella*. Moreover, the composition of the uterine and vaginal microbiota changes in a synchronized manner, with stable bacterial abundance correlations between the two sites in both healthy and diseased states. Importantly, the proportion of microbial translocation from the vagina to the uterus is significantly higher in women with endometritis, indicating a strong association between vaginal microbial migration and the disease state (31). In summary, we hypothesize that an imbalance in vaginal microorganisms may lead to endometritis. Additionally, the significant increase in pro-inflammatory cytokines in the endometrium of patients with uterine fibroids suggests that endometrial inflammation may contribute to the initiation and progression of uterine fibroids.

Although *Lactobacillus* vaginalis is beneficial for reproductive health, its decline is associated with many diseases, such as human papillomavirus infection and cervical cancer, endometriosis, infertility, and endometrial adhesion (32–35). However, an increase in *Lactobacillus* is also not conducive to reproductive health, and studies have shown that an excessive increase can lead to excessive acid in the vaginal environment, which damages vaginal epithelial cells, leading to cell lysis and inflammation (36). A high abundance of *Lactobacillus* is associated with assisted reproductive failure and tubal infertility (37). The *Lactobacillus*-dominated vaginal microbiota may increase the risk of preterm birth through local tissue inflammation and cervical integrity (37). In addition, hyperplasia of vaginal *Lactobacillus* may lead to an imbalance of the vaginal microbiota, increase the risk of persistent HPV infection and be associated with the development of invasive cervical cancer (33). Studies have shown that *Lactobacillus* is *increased* in the uterine fluid of endometriosis patients (38), and the related mechanism may be related to an increase in proinflammatory cytokines (39). This may be due to an inflammatory response triggered by an imbalance in the microbiome.

Therefore, the vaginal microbiome needs to be balanced to maintain reproductive health. Although the vaginal microbiota is dominated by *Lactobacillus*, its reduction and excessive increase are associated with inflammation and gynecological diseases, indicating that *Lactobacillus* needs to be moderate and that too many and too few *Lactobacillus* strains are not conducive to female reproductive health.

*Atopobium* is a gram-positive facultative anaerobic bacterium, and its increase has been associated with bacterial vaginosis, chronic endometritis, pelvic inflammatory disease, and infertility (32, 40–43). In bacterial vaginosis, *Atopobium* is an important pathogen that often forms biofilms in the vagina with other bacteria; this biofilm structure increases bacterial resistance and can lead to treatment failure and relapse (44, 45). In addition, *Atopobium* may also play a role in endometrial cancer (46, 47). Multiple studies have shown that vaginal microbiome diversity in patients with uterine fibroids is not significantly different from that in healthy women (27, 48). Studies have shown that alpha diversity is negatively correlated with the number of uterine fibroids. An increase in individual bacterial flora, such as *Erysipelatoclostridium*, *Mucispirillum*, and *Finegoldia* and *Erysipelotrichaceae* UCG-003 and *Sporolactobacillus*, was also detected in the vaginas of patients with uterine fibroids (48). Our study revealed that there was no significant difference in the diversity or complexity of the vaginal microbiota between patients with uterine fibroids and healthy women, but the abundance of *Atopobium* decreased. At present, there is no literature reporting the relationship between *Atopobium* and uterine fibroids, but studies have shown that *Atopobium* is completely absent in the vagina and cervix of patients with endometriosis (49). Although the mechanism of action of *Atopobium* in diseases of noninfectious etiology is unclear, it can promote infection with *Porphyromonas*, which can exist inside cells and disrupt cellular regulatory functions, ultimately leading to carcinogenic triggers (47). Whether this association is causal is unclear. Uterine fibroids are benign tumors, and from a different point of view, the absence of *Atopobium* may be related to the occurrence of uterine fibroids, indicating that an imbalance in the vaginal microecology may promote the occurrence and development of uterine fibroids. On the other hand, Current studies have unequivocally established that uterine fibroids are estrogen-dependent conditions (30, 50). Recent research analyzing the composition of the vaginal microbiome in postpartum and postmenopausal women revealed a significant increase in *Atopobium* species in states of low estrogen, suggesting a negative correlation with estrogen levels (51). Based on this, we hypothesize that patients with uterine fibroids may exhibit a reduction in *Atopobium* levels due to their elevated estrogen levels or heightened sensitivity to estrogen.

There was no obvious change trend among the groups of microbial function prediction analysis, which may be due to the small sample size. We will continue to include new samples for analysis and comparison in the future.

## Conclusion

In summary, although the microbiome diversity among the different groups was not significantly different, we detected a significant increase in vaginal *Lactobacillus* in patients with ovarian cysts and a significant decrease in vaginal *Atopobium* in patients with uterine fibroids, providing novel clues for deciphering the mechanisms of some benign gynecological diseases.

## Data Availability

The datasets presented in this study can be found in online repositories. The names of the repository/repositories and accession number(s) can be found below: https://www.ncbi.nlm.nih.gov/, SUB14859751.
